# Thermodynamic and Spectroscopic Studies of the Complexes Formed in Tartaric Acid and Lanthanide(III) Ions Binary Systems

**DOI:** 10.3390/molecules25051121

**Published:** 2020-03-03

**Authors:** Michal Zabiszak, Martyna Nowak, Zbigniew Hnatejko, Jakub Grajewski, Kazuma Ogawa, Malgorzata T. Kaczmarek, Renata Jastrzab

**Affiliations:** 1 Faculty of Chemistry, Adam Mickiewicz University in Poznan, 61-614 Poznan, Poland; martynan@amu.edu.pl (M.N.); zbychuh@amu.edu.pl (Z.H.); kgraju@amu.edu.pl (J.G.); gosiat@amu.edu.pl (M.T.K.); renatad@amu.edu.pl (R.J.); 2 Institute for Frontier Science Initiative, Kanazawa University, Kakuma-machi, Kanazawa, 920-1192 Ishikawa, Poland; kogawa@p.kanazawa-u.ac.jp

**Keywords:** lanthanide(III) ions complexes, tartaric acid, potentiometric measurements, spectroscopic studies

## Abstract

Binary complexes of tartaric acid with lanthanide(III) ions were investigated. The studies have been performed in aqueous solution using the potentiometric method with computer analysis of the data for detection of the complexes set, determination of the stability constants of these compounds. The mode of the coordination of complexes found was determined using spectroscopy, which shows: Infrared, circular dichroism, ultraviolet, visible as well as luminescence spectroscopy. The overall stability constants of the complexes as well as the equilibrium constants of the reaction were determined. Analysis of the equilibrium constants of the reactions and spectroscopic data allowed the effectiveness of the carboxyl groups in the process of complex formation.

## 1. Introduction

Lanthanides (Rare Earth Elements—REEs) and their coordination compounds due to their luminescent properties play a crucial role in biological systems, especially in the diagnosis and monitoring of the progress of treatment of cancer diseases. Due to their luminescent properties showing a characteristic line emission after light absorption that is enhanced by the surrounding ligands [1,2]. The paramagnetic properties of lanthanide(III) ions (Ln(III)) and their complexes make them suitable for use as a contrast media for Magnetic Resonance Imaging (MRI). Particularly gadolinium(III) ion complexes have been found widely used in imaging diagnostics [3]. Emission of lanthanide(III) ions, e.g., Nd(III), Sm(III), or Yb(III) in the Near-infrared (NIR) region that is detected through animal tissue of considerable thickness, could also be used for imaging in vivo [4]. Additionally, lanthanide(III) compounds are used as antibacterial agents [5,6,7,8] and show very effective catalytic properties with high selectivity for hydrolytic cleavage or transesterification of RNA and as a substance promoting DNA cleavage [9,10,11].

According to the Hard and Soft Acids and Bases (HSAB) theory, lanthanide(III) ions prefer the complexation with ligand with donor atoms in the order: O>>N>S. Tartaric acid with O-donor atoms are potentially good ligands and can be assembled in a diverse arrangement as a chelating or bridging species, and coordination complexes of a mononuclear, binuclear, and polymeric type could be formed [12,13,14,15,16].

**Figure 1:**
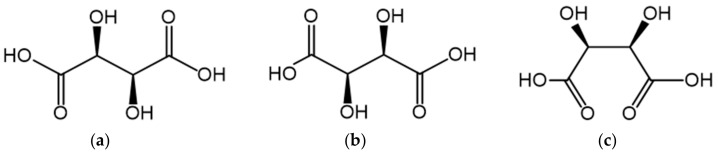
Formulae of tartaric acid: (**a**) Enantiomer L-(+); (**b**) enantiomer D-(-); (**c**) *meso* form.

Tartaric acid (Tar) is widely distributed in nature. It is present in many fruits in free form or as potassium, calcium, or magnesium salts [17]. Dicarboxylic tartaric acid containing two hydroxyl and two carboxyl groups and could be a tetradentate ligand [18]. Tartaric acid exists in three stereoisomers: The two enantiomers: (*R*,*R*)- also known as L-(+) or *(S,S)-* also known as D-(−)-, and the *meso* form (Figure 1). The (*R*,*R*)- and *(S,S)-* forms are optically active, the *meso* form, having a plane of symmetry within it, is optically inactive. The conformational preferences of tartaric acid [19,20] are essential for understanding the role of its properties in biological and chemical systems [21,22]. Only (*R*,*R*)-tartaric acid, its salts, and derivatives play an important role in pharmaceutical organic chemistry [23,24,25,26,27].These different forms of (*R,R*) and (*S,S*) tartaric acid affect the differences in the coordination ability of the ligand [28]. 

In spite of the fact that tartaric acid is a well-known molecule in the literature, it is difficult to find any potentiometric studies of complexes of lanthanide(III) ions with (*R,R*)tartaric acid. The main goal of the work was the determination of the type of interactions between lanthanide (III) ions/tartaric acid. These studies will be useful for more complicated systems, e.g., for the description of the impact of interactions in systems of lanthanide (III) ions/(S,S)tartaric acid with biogenic polyamines. We hope that the results of these studies will allow the detection of the level of polyamines, important in the diagnosis and monitoring of cancer diseases where the level of polyamines is of fundamental importance. 

In this paper, we present results of equilibrium and spectroscopic studies of the lanthanide(III) ions/(*R,R*)tartaric acid systems in aqueous solution. 

## 2. Results and Discussion

### 2.1. Equilibrium Study

In our previous studies, the coordination compounds of d- and f-metal ions with citric acid were described. It was found that citric acid complexes have a stoichiometry of metal:ligand 1:1 or 2:2 with three carboxyl groups as a potential coordination center [29]. In the current study in the binary system of tartaric acid with lanthanide(III) ions, complexes of stoichiometry metal:ligand 1:2 were detected (apart from lanthane(III) ions, where complexes with stoichiometry metal:ligand 1:1 were also found). The first step of our work included a determination of the protonation constants of (*R,R*) tartaric acid (the lack of convergence with the literature data results from other measurement conditions), Table 1. 

The potential coordination centers of the tartaric acid molecule are the two carboxyl groups. Tartaric acid occurs in solution in three forms H_2_Tar—protonated, HTar^-^—partly protonated, Tar^2-^— fully deprotonated. Full deprotonation of carboxyl groups occurs at low pH, Figure S1.

**Figure 2:**
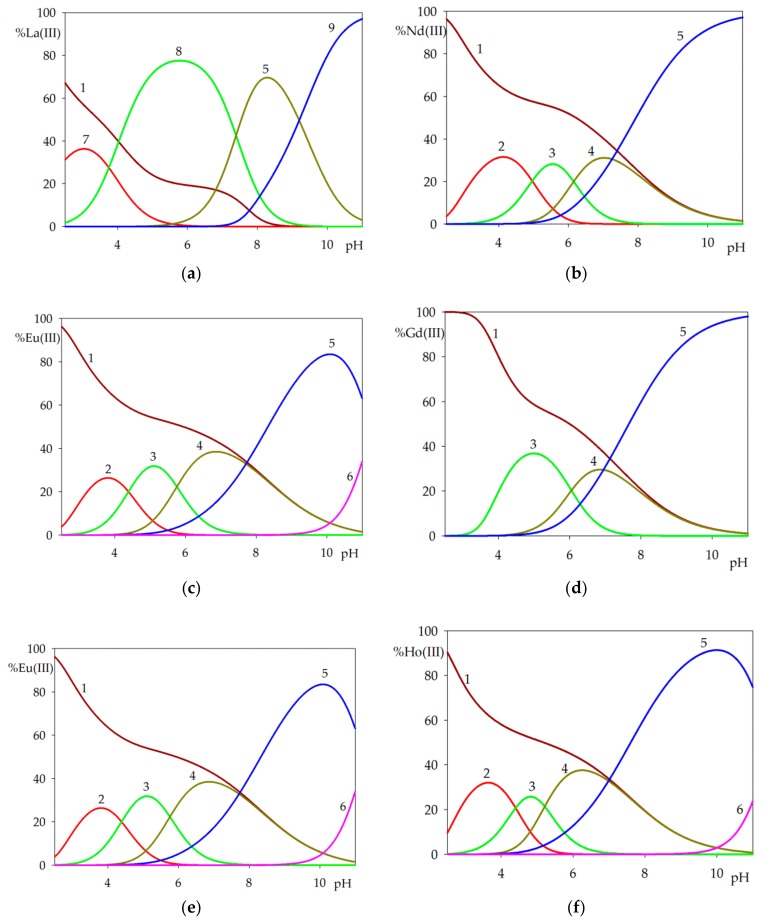

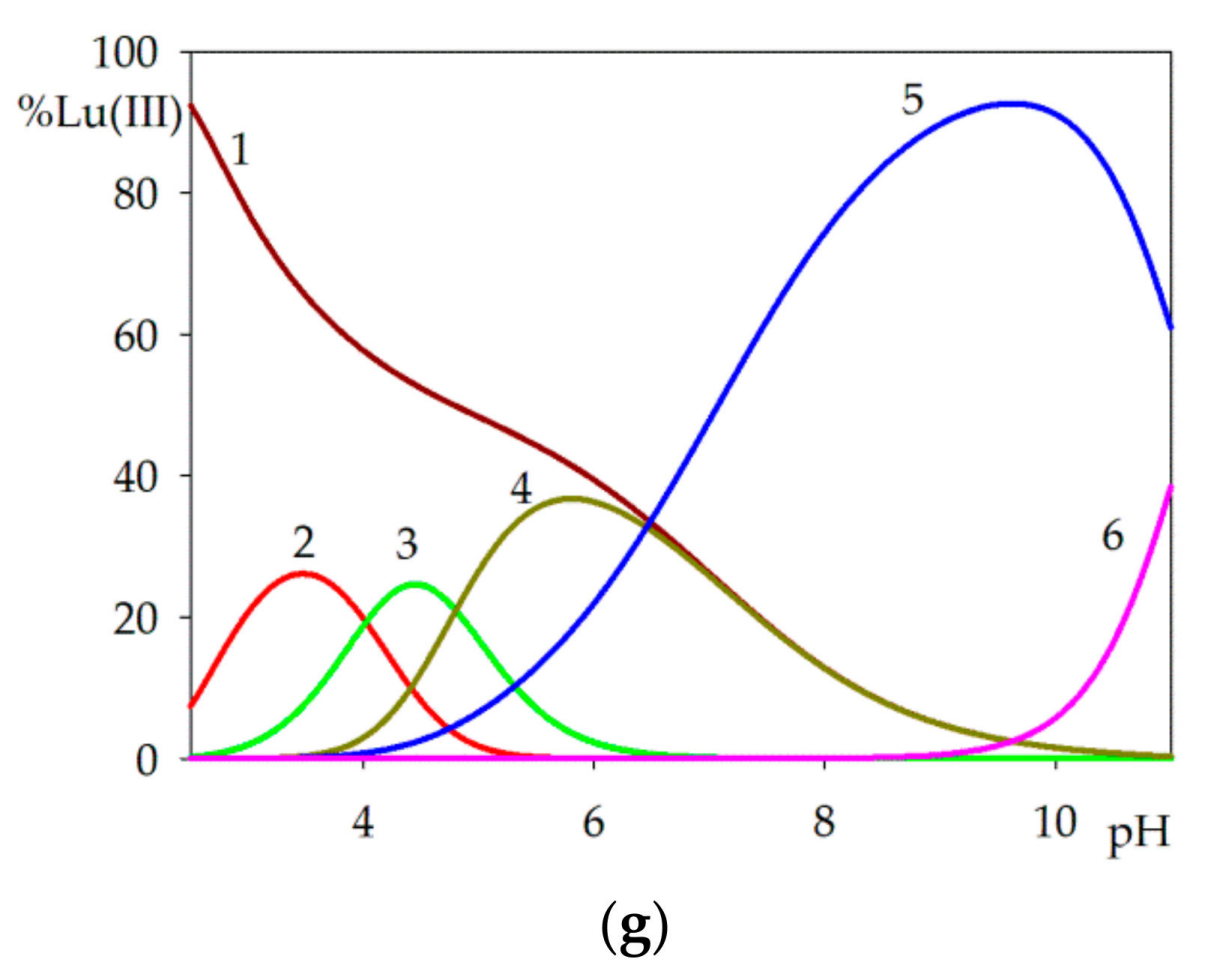
Distribution diagrams for the Ln(III)/Tar systems: (**a**) La(III)/Tar; (**b**) Nd(III)/Tar; (**c**) Eu(III)/Tar; (**d**) Gd(III)/Tar; (**e**) Tb(III)/Tar; (**f**) Ho(III)/Tar; (**g**) Lu(III)/Tar; 1 - Ln^3+^; 2 – Ln(HTar)(Tar); 3 – Ln(Tar)_2_; 4 – Ln(Tar)_2_(OH); 5 – Ln(Tar)(OH);6 – Ln(Tar)(OH)_2_; 7 – Ln(HTar); 8 – Ln(Tar); 9 - Ln(OH)_2_ the percentage of the species refers to lanthanide(III) ions; c_Ln(III) ion_ = 1 × 10^-3^ mol dm^-3^; c_Tar_ = 1 x 10^-3^ mol dm^−3^.

Computer analysis of the potentiometric data of the binary systems of (R,R)tartaric acid with lanthanide(III) ions (La(III), Nd(III), Eu(III), Gd(III), Tb(III), Ho(III), Lu(III)) has shown the formation of complexes. Complexes of Ln(HTar)(Tar), Ln(Tar)_2,_ and hydroxycomplexes of Ln(Tar)_x_(OH)_y_ types were identified in all systems, Figure 2. The composition and stability constants (logβ), as well as the equilibrium constants of the complexes formation (logK_e_), are presented in Table 2.

In the systems Ln(III)/Tar, where Ln(III)=Nd(III), Eu(III), Tb(III), Ho(III), and Lu(III), complexation begins with the formation of the protonated species Ln(HTar)(Tar) at a pH close to 4.00 and 20%–40% of free metal ions are involved; for the Gd(III)/Tar and La(III)/Tar systems the M(HTar)(Tar) complex is not formed in a detectable amount. For the Gd(III)/Tar system, no protonated species are formed, but for the La(III)/Tar system at pH 3.00, 40% of free metal ions introduced into the system are involved in complex La(HTar) formation. Equilibrium constants of Ln(HTar)(Tar) formation increases with the growth of the atomic number of lanthanides. The Ln(Tar)_2_ complex type is formed in all systems, and apart La(III)/Tar and occurs at a maximum concentration at a pH of about 5.00 and the fully deprotonated tartaric acid binds about 20%-30% of Ln(III) ions introduced into the system. The order of stabilities of Ln(Tar)_2_ species in terms of metal ions is Nd(Tar)_2_ 7.70 < Eu(Tar)_2_ 8.29 > **Gd(Tar)_2_ 7.65** < Tb(Tar)_2_ 8.51 < Ho(Tar)_2_ 8.83 < Lu(Tar)_2_ 9.05. As can be seen from the stability constants of the lanthanide complexes, the gadolinium complex has the lowest thermodynamic stability.

In the lanthanum(III) ion system, in contrast to the others, only monomeric complexes are formed. At pH 6.00, the dominant species is LaTar, which binds about 80% of metal ions introduced to the solution. At a pH of over 6.00 for all studied systems, three types of hydroxy complexes are formed: LnTar_2_(OH), LnTar(OH), and LnTar(OH)_2_. The first type, LnTar_2_(OH) is formed for Nd(III), Eu(III), Gd(III), Tb(III), Ho(III), and Lu(III) ions at pH 6.00–7.00 and binds about 30%–40% of lanthanide(III) ions. The order of equilibrium constants of Ln(Tar)_2_(OH) complexes according to the equilibrium Ln(Tar)_2_ + H_2_O ⇆ Ln(Tar)_2_(OH) + H^+^ is Nd(Tar)_2_(OH) 7.61 < Eu(Tar)_2_(OH) 8.00 > Gd(Tar)_2_(OH) 7.69 < Tb(Tar)_2_(OH) 8.15 < Ho(Tar)_2_(OH) 8.56 < Ln(Tar)_2_(OH) 8.96. The equilibrium constants of these complexes increase similarly for Ln(Tar)_2_(OH) complexes, Table 3. The next type, LnTar(OH), is formed at pH 10.00-11.00 and binds nearly 85%–95% of La(III), Nd(III), Eu(III), Gd(III), Tb(III), Ho(III), and Lu(III) ions. The last one complex type, LnTar(OH)_2,_ formed only for Eu(III), Tb(III), Ho(III), and Lu(III) ions at a pH of more than 11.00 and binds 20%–40% of metal ions. Equilibrium constants of the Ln(Tar)(OH)_2_ complexes as well as the other one show that species of tartaric acid formed with gadolinium(III) ions shows the lowest equilibrium constants of complex formation (Table 3) that was observed and described earlier and was called a *gadolinium break* [32,33].

### 2.2. Spectroscopy Studies

#### 2.2.1. IR Spectroscopy

**Figure 3:**
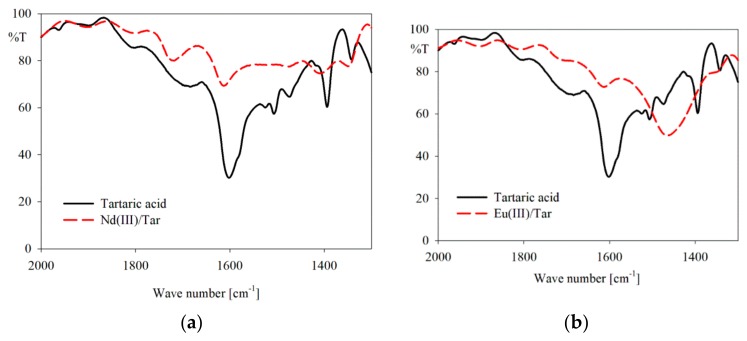
IR spectra of selected systems: (**a**) Nd(III)/Tar; (**b**) Eu(III)/Tar.

The IR spectra of the lanthanide(III) ion complexes confirm the involvement of the carboxyl groups of tartaric acid in complexes formation (Figure 3 and Figure S2). A reduction of the intensity or shift of the bands assigned to the stretching vibrations of C=O bonds and stretching vibrations of the C-O bonds were observed. The IR spectra were recorded at a pH of about 5.00 of Ln(Tar)_2_ dominance apart from the complex of La(III). 

#### 2.2.2. UV-Vis Spectroscopy

**Figure 4:**
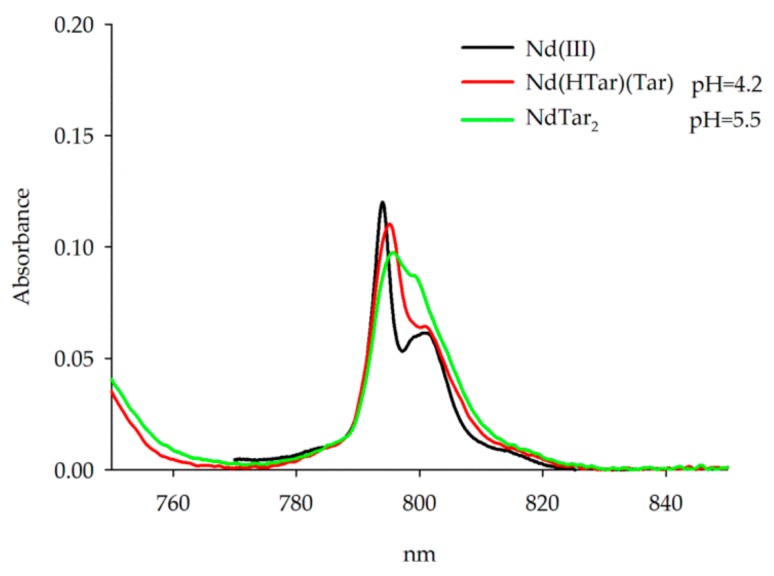
The absorption spectrum of Nd(III) ion in the system Nd(III)/Tar in the range of the transition ^4^I_9/2_ – ^2^H_9/2_.

The UV-Vis spectra were only recorded for the system Nd(III)/Tar at the pH values of domination of individual species in the range of the hypersensitive transition ^4^I_9/2_ – ^2^H_9/2_. The changes in the coordination sphere of Nd(III) ions affects the intensity of certain transitions in the absorption spectra of these ions, while the intensity of the remaining bands changes slightly [34,35,36]. The character of the absorption bands in the range of the hypersensitive transitions (^4^I_9/2_–^2^H_9/2_) allows confirmation of the formation of Nd(III) complexes, Figure 3 [37]. However, due to the weak nature of f-f transitions, the sensitivity of the absorption measurements was limited. The Nd(III) ion was chosen for the studies because it had the highest molar absorption coefficient, ε, in the whole lanthanide series [38]. The UV-Vis spectra covering the range assigned to this transition recorded for the solutions at pH values corresponding to the maximum concentration of individual complexes are presented in Figure 2b and Figure 4.

The spectra were recorded only at pH 4.20 and 5.50 due to the occurrence of the precipitate at higher pH values. The recorded spectra had different shapes and a shift of the maximum absorption from 794 to 795–796 nm (Figure 4, Table 3). 

The shifts of the maximum absorption and reduction of the intensity of bands revealed gradual deprotonation, depending on the pH value of tartaric acid. The changes of bands also indicated modification of the coordination sphere of the Nd(III) ion. An increased pH to 5.50 resulted in the disappearance of the band at 800.85 nm and a reduction in the intensity of the band at 794 nm [39].

#### 2.2.3. Luminescence Spectroscopy

**Figure 5:**
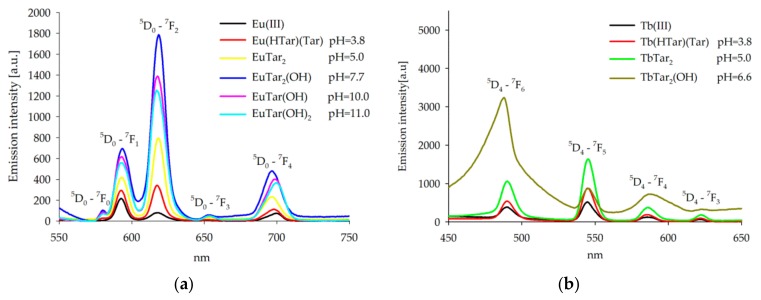
Changes in the emission spectra of the systems: (**a**) Eu(III)/Tar at pH 3.8, 5.0, 7.7, 10.0, and 11.00; (**b**) Tb(III)/Tar at pH 3.8, 5.0, and 6.6.

The emission spectra for Eu(III)/Tar and Tb(III)/Tar systems were recorded in the range of 450 to 750 nm using a few excitation wavelengths at the pH values of domination of the species. No emission was observed from the solvent or from the free tartaric acid. Particularly interesting were the bands corresponding to Ln(III) ion transitions in the 4f shell. The split of the 4f shell by an external field of ligands was very small. Thus the corresponding bands observed in the absorption and emission spectra of Ln(III) ions were narrow and sharp. As the type of ligand influences the bands corresponding to the f-f transitions in lanthanide ions, the absorption and luminescence spectroscopies were important in studies of the systems containing lanthanide(III) ions [37,40]. The studies of the emission of Eu(III) and Tb(III) complexes with tartaric acid in solution were measured at the pH at which the maximum concentration of the particular type of complexes occurred. The maxima of Eu(III) and Tb(III) emission in complexes with tartaric acid were found to appear at the same wavelengths as those of the respective uncomplexed ions, Figure 5.

For Eu(III)/Tar systems, the emission spectra were recorded for pH values from 3.80 to 11.00, while for Tb(III)/Tar systems, the emission spectra were recorded only for pH values 3.80, 5.00, and 6.60 because of precipitate formation at higher pH. 

For the systems containing Eu(III) ions, the emission begins from the nondegenerate ^5^D_0_ excited level and ends at the energy level ^7^F_j_ (j = 0, 1, 2, 3, 4) of the ground state, Figure 5. A strong emission was observed in the regions corresponding to the transitions ^5^D_0_-^7^F_1_, ^7^F_2_, while a medium-strong emission was found for the transition ^5^D_0_-^7^F_4_ during excitation with λ = 394 nm. An increase in the pH value of the studied Eu(III)/(Tar) systems was caused by increasing the intensity of the two bands (^5^D_0_-^7^F_1_, ^7^F_2_). The effect was particularly pronounced for the band at λ=618 nm. The ratio η=I_em_618/I_em_593 was determined and it was found that the excitation values for the studied systems were higher (Table 4) than those for the aqua complex Eu(H_2_O)_9_^3+^ (the value of η is 0.40). This observation shows that the water molecules in the internal coordination spheres of the central lanthanide ion are gradually replaced with molecules of tartaric acid during the complexation reaction [41].

Changes in the intensity of emission were observed for the Tb(III)/Tar system and to the emission corresponding to the transitions from ^5^D_4_ level to ^7^F_j_ (j = 6, 5, 4, 3), Figure 5b. 

The most intensive emission in the region 540–560 nm for Tb/Tar systems that corresponded to the transition ^5^D_4_-^7^F_5_ was observed for complex Tb(Tar)_2_. Whereas for complex Eu(Tar)_2_(OH), the most intensive emission was observed in the region 470–510 nm. The intensity of emission of Tb(III) complexes with tartaric acid was comparable to the emission of Eu(III) complexes analogous. 

#### 2.2.4. Circular Dichroism

The CD and corresponding UV spectra were recorded for all Ln(III) systems with (R,R)-tartaric acid in the range of 185 to 350 nm at a concentration of 1 × 10^−3^ mol dm^−^^3^ that allows a direct comparison to be made with the results of titration measurements. The whole series of samples were measured at a pH on the basis of the results obtained from the computer analysis of potentiometric data.

Analysis of the conformation of free tartaric acid and complexes with lanthanide(III) ions were carried out based on the observation of Cotton effects derived from the n-π* electron transitions of carboxylic groups. Tartaric acid in aqueous solutions exhibits trans conformation with a single negative Cotton effect Δε = −4 around 210–215 nm and its simple divalent salts show two negative Cotton effects Δε = −4 at 193 nm and -2.5 at around 211 nm. It should be mentioned that CD spectra of tartaric acid change their shape at different pH, Table 5.

**Figure 6:**
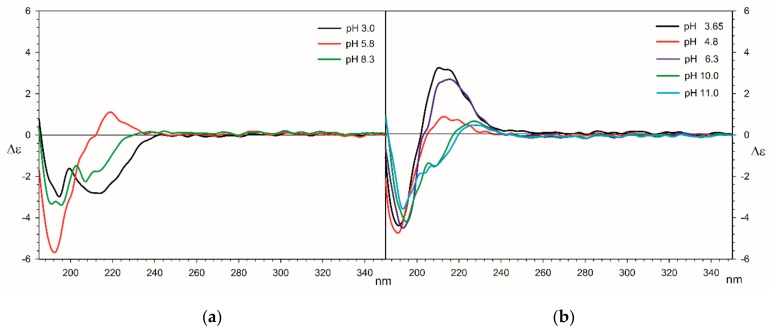
The Circular dichroism (CD) spectra of the systems (a) La/Tar; (b) Ho/Tar.

At low pH values, in the CD spectra, a positive cotton effect located in the 210-215 nm range is observed. It can be seen that tartaric acid is partially deprotonated, and two molecules of this acid coordinate to lanthanide(III) ions. The positive cotton effect at 219 nm indicates the complexation of the tartaric acid and confirms the results of the potentiometric measurements. The increase in the pH value caused a lowering and disappearance of CD effects. This indicates that the conformation of tartaric acid is changing as a result of the replacement of the acid molecules by hydroxyl groups in the coordination sphere of lanthanide(III) ions (Figure 6 and Figure S3). For all the studied systems at pH 11.00, trans conformation predominates because of electrostatic repulsions of negatively charged carboxylates and the formation of hydroxocomplexes. The different properties of the cotton effect on lanthanum(III) systems are caused by the bonding of only one tartaric acid molecule (other lanthanide (III) ions are bonded to two molecules of tartaric acid).

## 3. Materials and Methods

### 3.1. Materials

(R,R)-tartaric acid (Tar) was purchased from the Sigma Chemical Company. Lanthanide(III) nitrates and chlorides (La(III), Nd(III), Eu(III), Gd(III), Tb(III), Ho(III), and Lu(III)) were obtained from the Aldrich Chemical Company. All chemicals were used without further purification. The concentrations of solutions of lanthanide(III) ions were determined by inductively coupled plasma optical emission spectrometry (ICP OES). Demineralized carbonate-free water (conductivity 0.055 μS) was used to prepare all solutions. 

### 3.2. Equilibrium Study

Potentiometric titrations were carried out using a Titrando 905 Metrohm (Herisau, Switherland) equipped with an autoburette using an i-electrode Metrohm 6.0280.300 calibrated in terms of hydrogen ion concentration [42,43,44,45,46,47]. The pH-meter indication was corrected using 2 standard buffer solutions of pH 4.00 and pH 9.22 at 20 ± 1 °C. All potentiometric titrations were performed in an inert gas atmosphere (helium—ultra high purity). Experiments were performed at a constant ionic strength of 0.1 M (KNO_3_) with a temperature of 20 ± 1 °C, in the pH range from 2.50 to 10.50, using as a titrant CO_2_-free NaOH (0.1885 M). The studies were carried out at the metal:ligand ratios of 1:1 and 1:2 (the concentration of tartaric acid in all systems was 0.001M). For each studied system, 8 or more titrations were performed, and 150–350 points from each titration curve were used for computer analyses. The protonation constants of the tartaric acid and stability constants of the complexes were determined using the Hyperquad 2003 computer program [31,42,43,44]. The calculations allowed the determination of the types (stoichiometry) and thermodynamic stability of the complexes formed in the systems studied. The calculation procedure was started with the simplest hypothesis, and then the models were expanded to include progressively more species, after which the results were scrutinized to eliminate the species rejected by the refinement processes. The stability constants were evaluated for the equilibria: pLn + qH + r(Tar) ⇆ Ln_p_H_q_(Tar)_r_, where Ln=lanthanide ion (charges were omitted for simplicity), and calculated in the following equation: logβ = [Ln_p_H_q_Tar_r_]/[M]^p^[H]^q^[Tar]^r^. Hydrolysis constants of lanthanide(III) ions were taken into account in the computer analysis of potentiometric data, Table 6. The pK_w_ value determined was 13.89. The correctness of the model was confirmed by the verification of the results obtained [46,48,49]. The distribution diagrams of particular systems were obtained by the HySS (Hyperquad Simulation and Speciation) program [42].

### 3.3. Spectroscopy Studies

Infrared spectra were obtained with an ISS 66 v/S Bruker spectrometer (Ettlingen, Germany) (resolution of 2 cm^−1^), in the cells with Si windows (thickness 100 µm), and peak positions were reported in cm^−1^. The samples were prepared by dissolving: Tartaric acid and appropriate lanthanide(III) salts in D_2_O, and the concentration of metal ions was 0.25 M and a molar ratio of metal:ligand 1:1. The pH was adjusted by adding a deuterated acid DCl or base NaOD and corrected according to the formula pD = pH meter readings + 0.40 [50]. The Ultraviolet-visible spectra were obtained with an Evolution 300 UV-VIS spectrometer Thermo Fisher Scientific (Bremen, Germany) (resolution 0.2 nm) using a quartz cell with a 1 cm path length. The spectrum for the system Nd(III)/Tar was recorded with Shimadzu UV-2401PC (resolution 0.05 nm). The samples were prepared in water at the metal:ligand ratio 1:1, and the metal ion concentration was 0.05 M. Ultraviolet spectra for luminescence spectroscopy were recorded with a Cary 300Bio (Varian) (Middelburg, Netherlands) spectrophotometer, while emission spectra were measured on a Jasco 8500 spectrofluorophotometer using 2.5/2.5 nm slit widths. Samples were excited at 395 nm for Eu(III) systems and 370 nm for Tb(III) systems. The absorbance of the solutions at the excitation wavelength of 395 nm was <0.2 absorbance units. The samples were prepared in ultrahigh quality water. Water was deionized and purified using a Simplicity Ultrapure Water System (Millipore). Circular dichroism and correlated UV spectra were recorded on a JASCO J810 spectropolarimeter (Japan). All spectra were recorded in the nitrogen gas in the range of 185 to 350 nm and were accumulated with 8 scans. The concentrations were of 1 × 10^−3^ M, and the optical path length was 0.5 mm. All spectra were obtained at room temperature. 

## 4. Conclusions

The formation of complexes of lanthanide ions with (*R,R*)tartaric acid has been established. The formation of Ln(HTar), Ln(Tar), Ln(Tar)(OH), Ln(Tar)(OH)_2_, Ln(HTar)(Tar), Ln(Tar)_2_, Ln(Tar)_2_(OH) type complexes was confirmed. The stability constants as well as of these complexes increase with a decreasing ionic radius of lanthanide(III) ions, but for gadolinium(III) ion complexes, the lowest equilibrium constants of complex formation were found, known as a *gadolinium break*. The order of stabilities of the Ln(Tar)_2_ species in terms of metal ions is Nd(Tar)_2_ 7.70 < Eu(Tar)_2_ 8.29 > **Gd(Tar)_2_ 7.65** < Tb(Tar)_2_ 8.51 < Ho(Tar)_2_ 8.83 < Lu(Tar)_2_ 9.05 and the order of stability constants of Ln(Tar)_2_(OH) complexes types is similar. The photoluminescence of Tb(III), Eu(III) and their complexes with (R,R)tartaric acid was also investigated. The increase of pH values changes the coordination sphere of the lanthanide ion and water molecules are removed from this sphere. Additionally, CD spectroscopy confirmed that lanthanum(III) ions contain only one tartaric acid molecule in the internal coordination sphere. This paper is the first stage for further discussion on more complicated systems containing other bioligands, such as nucleosides, nucleotides, as well as polyamines.

## Figures and Tables

**Table 1:** Protonation constants of tartaric acid (standard deviation are given in parenthesis).

Species	Overall Protonation Constants (log*β*) [30,31]		Reactions	Successive Protonation Constants (log*K_e_*)
H_2_Tar	7.50(4)	6.78	HTar^-^ + H^+^ ⇆ H_2_Tar	2.59
HTar	4.91(4)	3.96	Tar^2-^ + H^+^ ⇆ HTar^-^	4.91

**Table 2:** Overall stability constants (logβ) and equilibrium constants of formation (logK_e_) of binary complexes in the system of Ln(III)/tartaric acid (standard deviation are given in parenthesis).

Species	Overall Stability Constants (log*β*)	Reaction	Equilibrium Constants (log*K_e_*)
La(HTar)	8.10(9)	La^3+^ + H(Tar) ⇆ La(HTar)	3.19
La(Tar)	4.35(5)	La^3+^ + Tar ⇆ La(Tar)	4.35
La(Tar)(OH)	−3.04(5)	La(Tar) +H_2_O ⇆ La(Tar)(OH) + H^+^	6.36
Nd(HTar)(Tar)	12.66(3)	Nd^3+^ + H(Tar) + Tar ⇆ Nd(HTar)(Tar)	7.75
Nd(Tar)_2_	7.70(7)	Nd^3+^ + 2Tar ⇆ Nd(Tar)_2_	7.70
Nd(Tar)_2_(OH)	1.55(5)	Nd(Tar)_2_ + H_2_O ⇆ Nd(Tar)_2_(OH) + H^+^	7.61
Nd(Tar)(OH)	−2.89(4)	Nd^3+^ + Tar + H_2_O ⇆ Nd(Tar)(OH) + H^+^	10.87
Eu(HTar)(Tar)	12.68(4)	Eu^3+^ + H(Tar) + Tar ⇆ Eu(HTar)(Tar)	7.77
Eu(Tar)_2_	8.29(5)	Eu^3+^ + 2Tar ⇆ Eu(Tar)_2_	8.29
Eu(Tar)_2_(OH)	2.53(4)	Eu(Tar)_2_ + H_2_O ⇆ Eu(Tar)_2_(OH) + H^+^	8.00
Eu(Tar)(OH)	−2.60(2)	Eu^3+^ + Tar + H_2_O ⇆ Eu(Tar)(OH) + H^+^	11.16
Eu(Tar)(OH)_2_	−13.87(6)	Eu(Tar)(OH) + H_2_O ⇆ Eu(Tar)(OH)_2_ + H^+^	2.50
Gd(Tar)_2_	7.65(4)	Gd^3+^ + 2Tar ⇆ Gd(Tar)_2_	7.65
Gd(Tar)_2_(OH)	1.58(7)	Gd(Tar)_2_ + H_2_O ⇆ Gd(Tar)_2_(OH) + H^+^	7.69
Gd(Tar)(OH)	−2.94(5)	Gd^3+^ + Tar + H_2_O ⇆ Gd(Tar)(OH) + H^+^	10.82
Tb(HTar)(Tar)	12.77(4)	Tb^3+^ + H(Tar) + Tar ⇆ Tb(HTar)(Tar)	7.86
Tb(Tar)_2_	8.51(4)	Tb^3+^ + 2Tar ⇆ Tb(Tar)_2_	8.51
Tb(Tar)_2_(OH)	2.89(4)	Tb(Tar)_2_ + H_2_O ⇆ Tb(Tar)_2_(OH) + H^+^	8.15
Tb(Tar)(OH)	−2.19(2)	Tb^3+^ + Tar + H_2_O ⇆ Tb(Tar)(OH) + H^+^	11.57
Tb(Tar)(OH)_2_	−13.30(5)	Tb(Tar)(OH) + H_2_O ⇆ Tb(Tar)(OH)_2_ + H^+^	2.66
Ho(HTar)(Tar)	13.23(4)	Ho^3+^ + H(Tar) + Tar ⇆ Ho(HTar)(Tar)	8.32
Ho(Tar)_2_	8.83(6)	Ho^3+^ + 2Tar ⇆ Ho(Tar)_2_	8.83
Ho(Tar)_2_(OH)	3.62(6)	Ho(Tar)_2_ + H_2_O ⇆ Ho(Tar)_2_(OH) + H^+^	8.56
Ho(Tar)(OH)	−1.69(3)	Ho^3+^ + Tar + H_2_O ⇆ Ho(Tar)(OH) + H^+^	12.07
Ho(Tar)(OH)_2_	−13.19(9)	Ho(Tar)(OH) + H_2_O ⇆ Ho(Tar)(OH)_2_ + H^+^	2.26
Lu(HTar)(Tar)	13.07(4)	Lu^3+^ + H(Tar) + Tar ⇆ Ho(HTar)(Tar)	8.16
Lu(Tar)_2_	9.05(5)	Lu^3+^ + 2Tar ⇆ Ho(Tar)_2_	9.05
Lu(Tar)_2_(OH)	4.25(6)	Lu(Tar)_2_ + H_2_O ⇆ Lu(Tar)_2_(OH) + H^+^	8.96
Lu(Tar)(OH)	−1.11(4)	Lu^3+^ + Tar + H_2_O ⇆ Ho(Tar)(OH) + H^+^	12.65
Lu(Tar)(OH)_2_	−12.31(7)	Lu(Tar)(OH) + H_2_O ⇆ Lu(Tar)(OH)_2_ + H^+^	2.57

**Table 3:** Spectroscopic data of systems Nd(III)/Tar.

Species	pH	λ_max_[nm]	ε [mol⋅dm^−3^⋅cm^−1^]
Nd(III)	4.2	794.00	12.0
		800.85	6.0
Nd(HTar)(Tar)	4.2	795.05	11.0
		801.14	6.4
Nd(Tar)_2_	5.5	795.70	9.7
		798.93	8.7

**Table 4:** The η = I_em_618/I_em_593 of binary complexes in Eu(III)/tartaric acid system.

Species	η = I_em_618/I_em_593
Eu(HTar)(Tar)	1.18
Eu(Tar)_2_	1.89
Eu(Tar)_2_(OH)	2.55
Eu(Tar)(OH)	2.28
Eu(Tar)(OH)_2_	2.22

**Table 5:** Δε values for all Ln(III)/Ta systems at different pH values.

La(III)/Tar	Nd(III)/Tar	Eu(III)/Tar	Gd(III)/Tar	Ho(III)/Tar	Tb(III)/Tar	Lu(III)/Tar
pH = 3.0 Δε (nm) −3.0 (195) −2.8 (213)	pH = 4.2 Δε (nm) −4.1 (194) 2.6 (215)	pH = 3.8 Δε (nm) −5.1 (193) 3.4 (210)3.4 (215)		pH = 3.65 Δε (nm) −4.3 (192) 3.2 (211)	pH = 3.8 Δε (nm) −5.3 (193) 4.4 (215)	pH = 3.8 Δε (nm) −3.0 (190) −0.5 (209) 0.5 (211) −0.3 (222)
pH = 5.8 Δε (nm) −5.7 (193) 1.1 (219)	pH = 5. 55 Δε (nm) −2.6 (194) 3.1 (215)	pH = 5.1 Δε (nm) −4.2 (193) 3.7 (212)	pH = 5.0 Δε (nm) −3.1 (194) 3.7 (210) 3.8 (215)	pH = 4.8 Δε (nm) −4.7 (191) 0.8 (213)	pH = 5.0 Δε (nm) −4.9 (194) 3.3 (217)	pH = 4.5 Δε (nm) −3.0 (194) 1.7 (225)
	pH = 7.0 Δε (nm) −2.3 (195) −1.5 (206) −0.4 (214) −1.4 (223)	pH = 6.8 Δε (nm) −4.6 (194) 1.1 (222)	pH = 6.8 Δε (nm) −2.7 (195) 3.9 (217)	pH = 6.3 Δε (nm) −4.5 (193) 2.7 (215)	pH = 6.6 Δε (nm) −5.4 (194) 3.5 (215)	pH = 5.8 Δε (nm) −2.8 (194) 2.4 (211) 2.6 (217)
pH = 8.3 Δε (nm) −3.3 (191) −3.4 (196) −2.2 (208)		pH = 10.0 Δε (nm) −3.8 (194) −1.9 (208) 1.1 (227)		pH = 10.0 Δε (nm) −4.2 (195) −1.5 (209) 0.7 (227)	pH = 9.9 Δε (nm) −4.5 (195) −2.3 (205) 1.2 (227)	pH = 9.7 Δε (nm) −2.9 (196) −1.9 (210) 0.5 (226)
	pH = 11.0 Δε (nm) −2.7 (194) −2.2 (205) −1.1 (210) 0.9 (228)	pH = 11.0 Δε (nm) −3.0 (192) −1.4 (206) 1.6 (229)	pH = 11.0 Δε (nm) −2.5 (194) −1.1 (226)	pH = 11.0 Δε (nm) −3.6 (194) −1.5 (209) −0.5 (228)	pH = 11.0 Δε (nm) −4.7 (194) −2.0 (208)	pH = 11.0 Δε (nm) −2.6 (192) −2.7 (198) −1.7 (210) 0.2 (232)

**Table 6:** Hydrolysis constants of lanthanide(III) ions (standard deviations are given in parenthesis).

	Overall Hydrolysis Constants (log*β*)		
	M(OH)	M(OH)_2_	M(OH)_3_
**La^3+^**		−15.54(1)	
**Nd^3+^**		−16.12(2)	−25.53(4)
**Eu^3+^**		−15.15(3)	−24.31(6)
**Gd^3+^**	−9.30(1)	−17.74(2)	−28.12(6)
**Tb^3+^**		−15.56(1)	
**Ho^3+^**		−15.62(1)	
**Lu^3+^**		−14.93(1)	

## Supplementary Materials

The following are available online at https://www.mdpi.com/1420-3049/25/5/1121/s1, Figure S1: Distribution diagram of protonation forms of tartaric acid cTar = 1 × 10^−3^ mol dm-3, Figure S2: IR spectra of studied systems: a)La(III)/Tar; b) Gd(III)/Tar; c) Tb(III)/Tar; d) Ho(III)/Tar; e) Lu(III)/Tar, Figure S3: The CD spectra of the systems: (a) Nd/Tar; (b) Eu/Tar; (c) Gd/Tar; (d) Tb/Tar; (c) Lu/Tar.
