# Supplementary material for: Thermodynamic and Spectroscopic Studies of the Complexes Formed in Tartaric Acid and Lanthanide(III) Ions Binary Systems
Source: Molecules. 2020 Mar 3;25(5):1121. doi: 10.3390/molecules25051121 (PMC7179146; doi:10.3390/molecules25051121)
Supplement: Supplementary file 1 [file molecules-25-01121-s001.pdf]

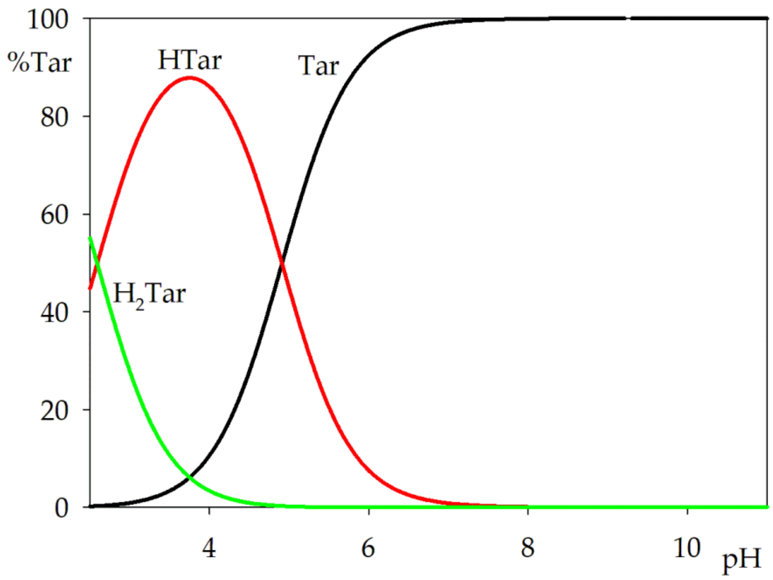

Figure S1. Distribution diagram of protonation forms of tartaric acid c_{Tar} = 1 x 10^{-3} mol dm^{-3}.

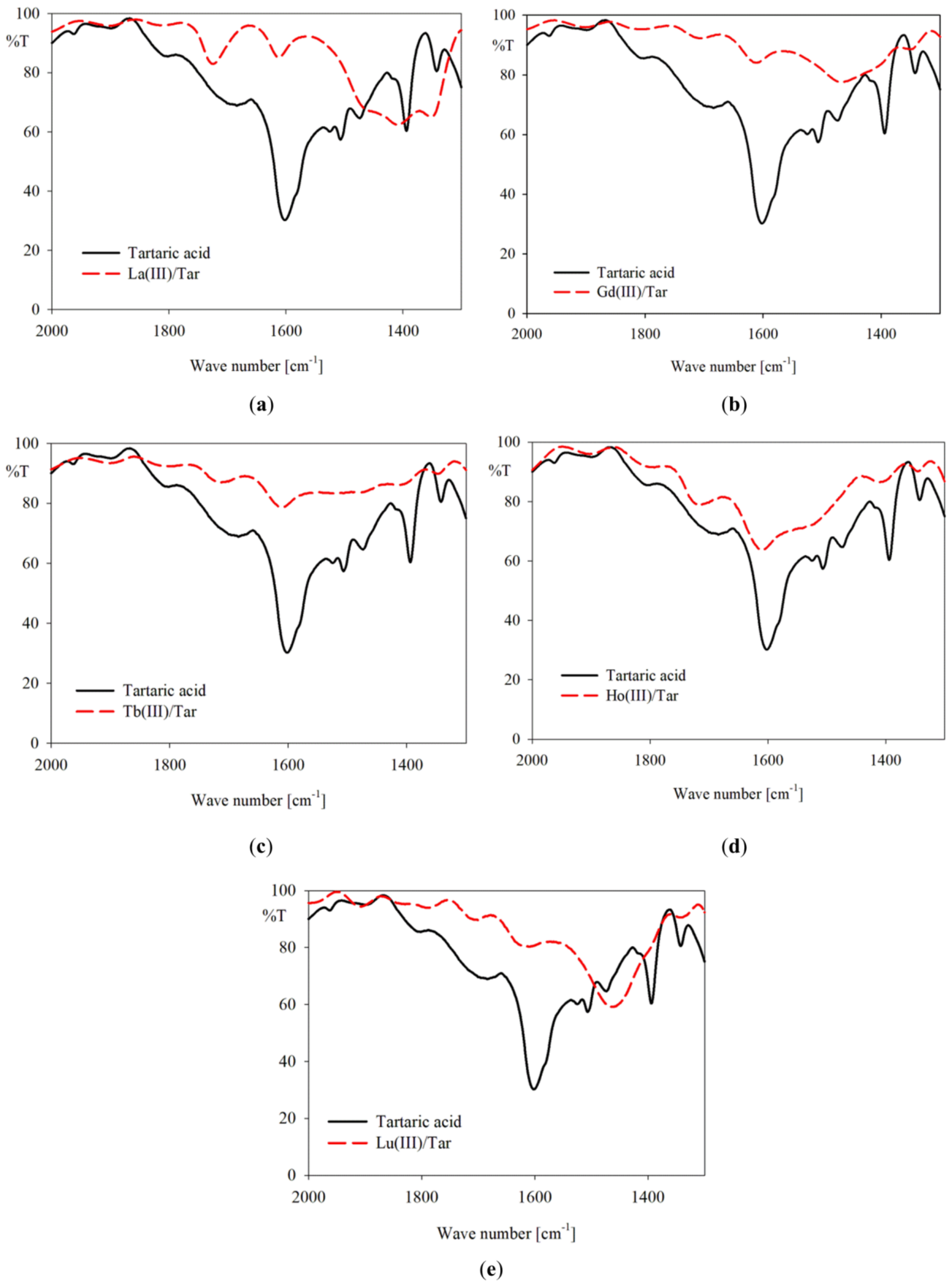

Figure S2. IR spectra of studied systems: (**a**) La(III)/Tar; (**b**) Gd(III)/Tar; (**c**) Tb(III)/Tar; (**d**) Ho(III)/Tar; (**e**) Lu(III)/Tar.

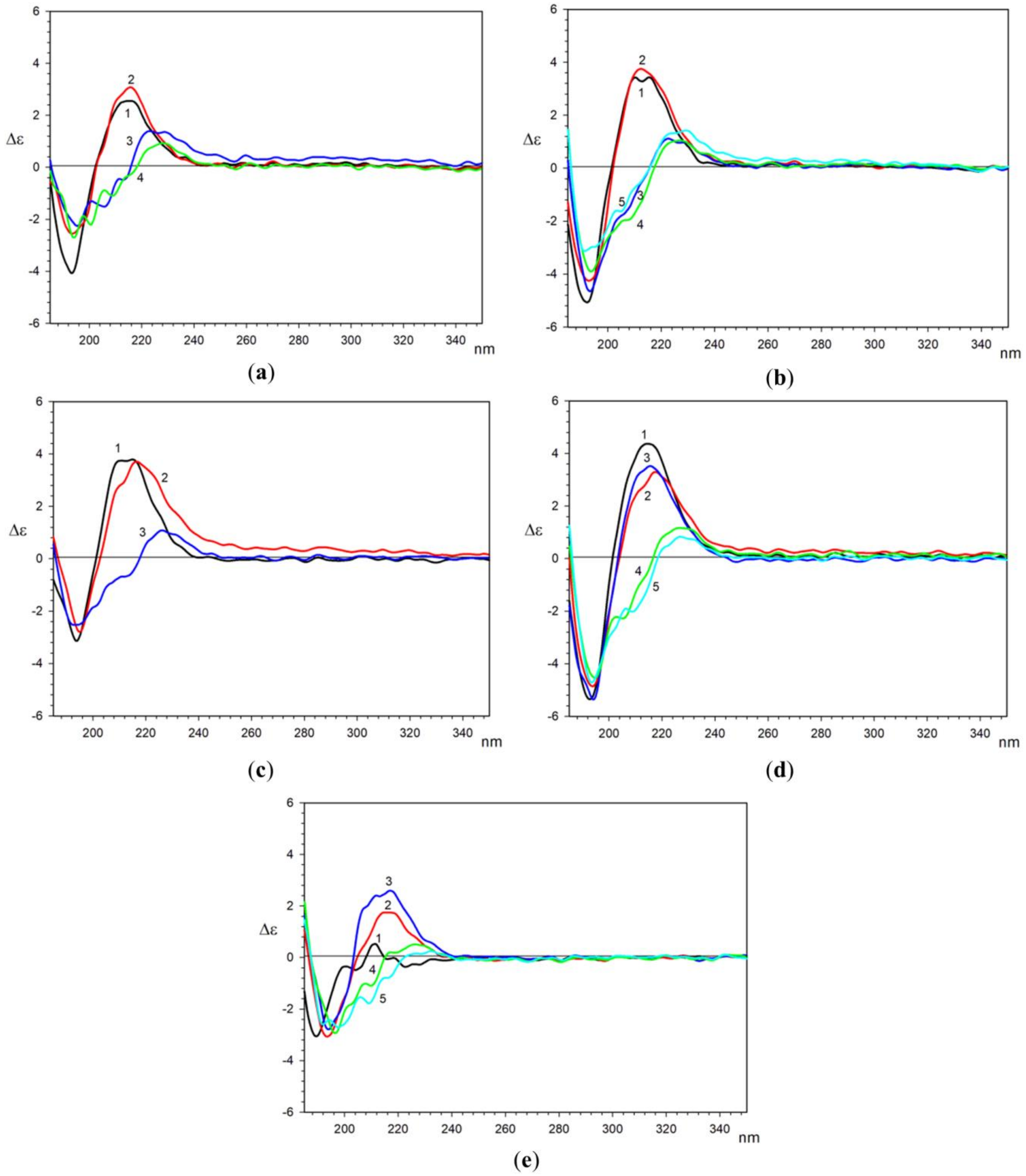

Figure S3. The CD spectra of the systems: (**a**) Nd/Tar: 1-pH=4.20, 2-pH=5.55, 3-pH=7.00, 4-pH=11.00; (**b**) Eu/Tar: 1-pH=3.80, 2-pH=5.10, 3-pH=6.80, 4-pH=10.00, 5-pH=11.00; (**c**) Gd/Tar: 1-pH=5.00, 2-pH= 6.80, 3-pH=11.00; (**d**) Tb/Tar: 1-pH=3.80, 2-pH=5.00, 3-pH=6.60, 4-pH=9.90, 5-pH=11.00; (**e**) Lu/Tar: 1-pH=3.80, 2-pH=4.50, 3-pH=5.80, 4-pH=9.70, 5-pH=11.00.
